# Seasonal Synchronization of Diapause Phases in *Aedes albopictus* (Diptera: Culicidae)

**DOI:** 10.1371/journal.pone.0145311

**Published:** 2015-12-18

**Authors:** Guillaume Lacour, Lionel Chanaud, Grégory L’Ambert, Thierry Hance

**Affiliations:** 1 EID Méditerranée, Montpellier, France; 2 Centre de Recherche sur la Biodiversité, Earth and Life Institute, Université catholique de Louvain, Louvain-la-Neuve, Belgique; University of Cincinnati, UNITED STATES

## Abstract

In temperate areas, population dynamics of the invasive Asian tiger mosquito *Aedes albopictus* are strongly affected by winter. The work we present here analyzes the adaptive synchronization of the diapause process in the wintry generation of *A*. *albopictus*, where the egg stage is exposed to adverse winter conditions. The seasonal pattern of egg laying activity of a French Mediterranean population of the Asian tiger mosquito was monitored weekly for 2 years with ovitraps. The field diapause incidence and the critical photoperiod (CPP, i.e. the maternal day length inducing diapause in 50% of the eggs), were determined by hatching experiments on the collected eggs. The period of diapause termination was estimated by a field survey of the first hatchings for both years. The CPP is equal to 13.5 hours of light and occurs in the field on the 25^th^ of August. Thus, it is on September 11^th^, 17 days after the CPP, that 50% of the eggs are in a prediapause stage in the field. The egg diapause rate increases rapidly during September, whereas the mean number of eggs laid decreases sharply after mid-September. Surprisingly, after having reached a peak of 95% at the end of September, from mid-October the diapause incidence declined and stayed below 50%. Indeed, both years the diapause initiates before the rapid decrease of the environmental temperature. This leaves a sufficient period of time to the complete development of one generation of *A*. *albopictus* with effective induction of diapause in the laid eggs. The very first larvae hatched were sampled both years in the first half of March. With 20 to 26 weeks in the egg stage and about 7 weeks in the larval stages, the first annual generation spends a long time in immature stages. On a practical point of view, this long development time represents a wide window for eggs and larvae control in early spring.

## Introduction

The Asian tiger mosquito *Aedes albopictus* (Skuse 1894) is an invasive species, extending its distribution range faster than any mosquito species during the last 30 years [1]. Native to South-East Asia, the first efficient establishment of *A*. *albopictus* in a temperate country outside its native area was recorded in Albania in 1979 [2]. In 1985 it started to invade the United States of America from Japan [3] and in 1990 Western Europe, especially Mediterranean areas, from the United States [4]. In both cases the species was imported as dormant eggs through used tires trade [3,5,6]. The vector competence of the Asian tiger mosquito was demonstrated for 27 arboviruses, but its role in field outbreaks was only proved for dengue, chikungunya and Zika viruses [7,8]. The risk of emergence or re-emergence of these diseases is relevant with regards to the European outbreak history. The most explosive and virulent dengue outbreak was recorded in 1927–28 in Greece, and was transmitted by another urban and anthropophilic vector, *A*. *aegypti* [9]. It is a topic of high concern with autochthonous cases of dengue fever reported in France repeatedly since 2010 [10,11,12] and in Croatia in 2010 [13]. Similarly, *A*. *albopictus* is responsible for a chikungunya outbreak in Italy in 2007 [14] as for indigenous transmissions in France in 2010 [15] and in 2014 [16]. However detailed field data on its phenology are still lacking, and particularly on the incidence of winter dormancy, to better understand its seasonal dynamics. These data are needed to assess the risk of mosquito-borne diseases transmission and to develop adequate vector control strategies in temperate areas.

The Asian tiger mosquito shows a remarkable ecological and physiological plasticity [7]. The environmental parameters of importance to define this species suitable habitat have been identified [17,18,19] as well as the epidemiological hazard [20]. In temperate areas, winter temperature is the major factor limiting the persistence of *A*. *albopictus* [21,22]. Temperate populations are able to withstand winter at the stage of egg diapause [1]. Diapause is a process of arrested development of the organism occurring before environmental conditions become harsh, permitting the optimal temporal adaptation of the species to its environment [23]. The seasonal dynamics of the diapause process is of prime importance to decipher its phenology. The terms and definitions of the diapause phases used here refer to the review of Koštál [24]. The facultative egg diapause is induced by maternal photoperiod [25]. Females are sensitive to short photoperiod at pupal and adult stages. They then produce a signal to trigger diapause preparation in their oocytes. Diapause initiation is effective once the embryo is fully developed [26]. The prediapause phase includes all events from diapause induction to diapause initiation. The diapause phase ends by the diapause termination, as the gradual reactivation of the eggs after winter. In many insects, the major environmental factors in diapause termination are low temperatures and an extended photoperiod [27]. Eggs will stay in a post-diapause quiescence after the diapause termination up until they experience a springtime hatching stimulus. As winter temperatures in Southern France are a strong constraint for the survival of this species, the invasive populations probably underwent a strong selection pressure. The adaptation of the photoperiodic induction of diapause in a particular area is known to happen quickly in *A*. *albopictus* [28]. Thus we expect that *A*. *albopictus* populations from the French Riviera are able to respond to the local photoperiodic conditions through a maternally induced diapause allowing them to avoid temporal harsh conditions.

The work presented here attempts to analyze the complete diapause process of the wintry generation of *A*. *albopictus*, from events occurring during their maternal oogenesis to the oviposition of their offspring. For this purpose, we isolated and quantified the effects of photoperiod on diapause incidence in a natural population. (1) Using individuals sampled in the field, we determined the critical photoperiod (CPP) of the population, i.e. the day/night ratio that the laying females must be exposed in order to induce diapause in 50% of the eggs laid. The CPP was compared to the field diapause incidence. (2) The period of dormancy termination was then estimated by a field monitoring of the first hatchings on both years. (3) Lastly, using a day degree model, the egg laying activity data of 2 consecutive sliding years were analyzed according to the phenology of diapause and climatic conditions.

## Materials and Methods

### Ethic statement

The animal facility of the ‘‘Entente Interdépartementale pour la Démoustication du littoral méditerranéen” has received accreditation from the French Ministry of Agriculture to perform experiments on live guinea pig (permit number B34-172-29) in appliance of the French and European regulations on care and protection of Laboratory Animals. Field survey was realized with permission from the Direction Générale de la Santé (DGS) of the French ministry in charge of health.

### Experimental field area

**Fig 1:**
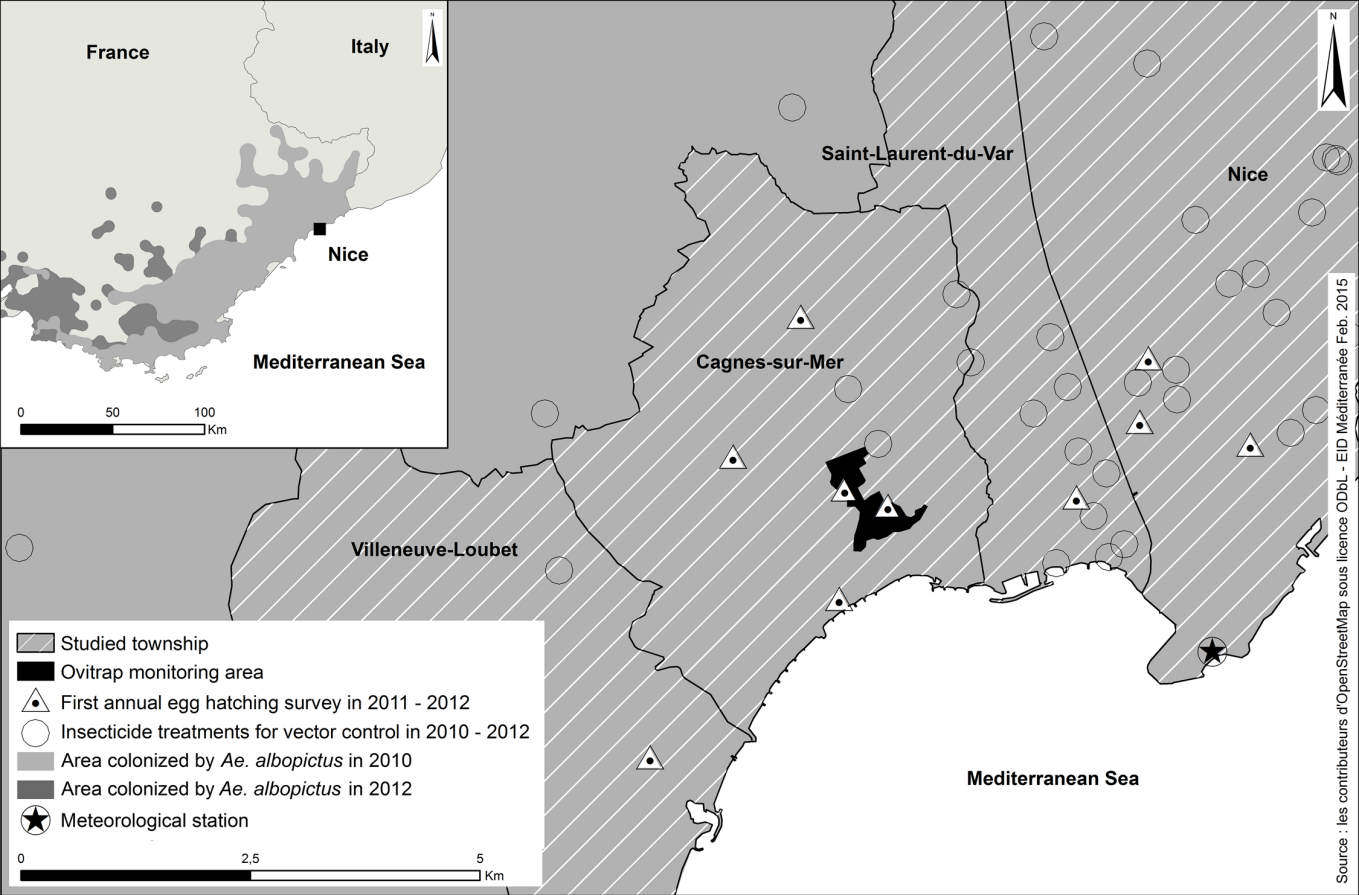
Experimental area for seasonal egg laying dynamics and first annual egg hatching monitoring of *Aedes albopictus* in Mediterranean France. The area monitored by ovitraps from summer solstice 2010 to summer solstice 2012 was computed as the surface of the smallest polygon including all traps with a buffer distance of 50 m (black area). This area was safe from outdoor insecticide spraying for vector control (circles). Ten infested places localized around the ovitraps network were inspected daily in March 2011 and 2012 to detect the first larvae of the year (triangles).

The diapause incidence survey was performed in the Alpes-Maritimes region, France, where *A*. *albopictus* is established since 2004 [29] (S1 Table). It is the only species of the subgenus *Stegomyia* present in France since the 1950’s eradication of *A*. *aegypti* [30]. An ovitrap network was installed at the summer solstice 2010 in Cagnes-sur-Mer (43°66'N, 7°6'E), a city colonized by the species since 2006 (Fig 1). A residential area mainly composed of individual houses with gardens was chosen for the monitoring. During our experimental period, no insecticide treatment was realized in the vicinity of the ovitraps, even though this experiment took place within the framework of the national anti-dissemination plan for chikungunya and dengue viruses [31]. Sites in the neighboring area were chosen to survey the spring larval development. Eggs collected on ovitraps up until July 26^th^ 2010, a period of the year not expected to induce diapause, were reared as progenitors (F0 generation) of laboratory mosquitoes.

### Diapause incidence

#### Experimental determination of diapause incidence and critical photoperiod

**Fig 2:**
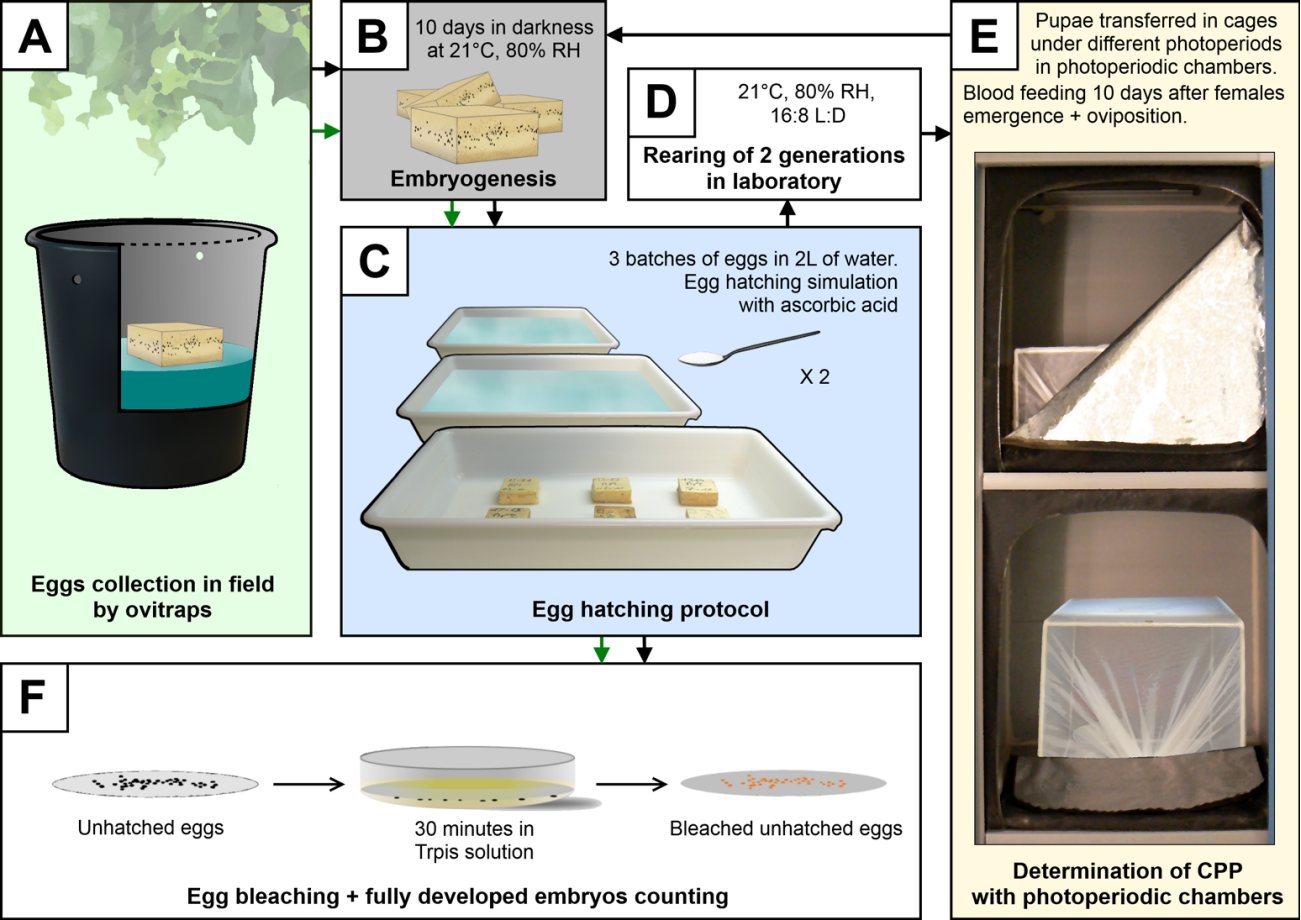
Protocol for the determination of egg diapause incidence in *Aedes albopictus*. The critical photoperiod (CPP) of the population is determined by an experiment including all steps in this order: A, B, C, D, E, B, C and F (black arrows). The survey of diapause incidence in the field follows steps A, B, C and F (green arrows).

The purpose of this experiment was to determine the photoperiod response of a sampled population of *A*. *albopictus*. Specifically, the objective was to calculate the critical photoperiod (CPP). Since day length is changing daily in the field, the CPP could hardly be deduced from observations and is more surely determined under fixed day length in the laboratory. Two generations of mosquitoes were reared at 21°C, 80% relative humidity and a light:darkness (L:D) 16:8 photoperiod (Fig 2). Eggs of F2 generation were stimulated to hatch under standardized conditions, and larvae were reared in batches of 250 larvae per pan of 1 liter tap water and fed with 1.75 g of milled dog food. Eleven days after egg hatching, pans containing fourth instar larvae with first pupae were transferred in cages in photoperiodic chambers. Three batches of larvae were used per photoperiodic conditions, as follows: 13:11, 13.25:10.75, 13.5:10.5, 13.75:10.25 and 14:10 L:D hours. Photoperiodic chambers consisted of windowless plastic boxes (65 x 65 x 40 cm) with a black-tissue zipper opening. Individual chambers were maintained at a constant temperature of 21.5°C and 80% relative humidity, using a fan-produced air flow and a periodic air dampening system made of a water pot stirred using an aquarium air-pump. Light cycle was computer controlled and generated with a 3 watts bar of 42 white LED per photoperiodic chamber. There was no light transition between photophase and scotophase. Adult mosquitoes were supplied with a 10% sucrose solution. Females were blood-fed on an anesthetized Guinea pig 10 days after emergence. Five days after blood feeding, each cage was provided with an oviposition cup containing water and a strip of paper. The paper was removed 5 days later and eggs were conserved in petri dishes in darkness during 10 days to allow complete embryo development [26].

In order to determine the egg diapause rate, the resulting batches of eggs were immersed in containers filled with 2 liters of water (Fig 2). A decrease in dissolved oxygen concentration was generated with the addition of 100mg of ascorbic acid per liter of water 30 minutes after flooding [32]. This powerful hatching stimulus suppressed egg quiescence [27]. This protocol was repeated one day later, after air drying the eggs during 2 hours, to ensure the efficiency of the hatching stimulation. After two consecutive hatching stimulations, hatched eggs were counted rather than hatched larvae, the latter being prone to mortality and more difficult to count (S1 Fig). To determine the viable state of unhatched eggs, they were first bleached in Trpiš solution [33] to allow embryo observation. Eggs were considered viable when the embryo presented pigmented ocelli, an egg burster and were without abnormal pigmentation or malformation [26]. Diapause rate was calculated with hatched and unhatched embryonated egg numbers:

Diapause incidence%=(embryonated unhatched eggs x100)/(embryonated unhatched eggs+hatched eggs)

#### Field survey of diapause incidence

Once collected in the ovitraps (see below), eggs were stored for at least of 10 days in a humid atmosphere under complete darkness at 21°C. In 2010, eggs collected in the same week were studied as a uniform batch. For increased accuracy, 2011’s eggs were weekly separated in three batches of 100 eggs minimum in order to obtain the standard deviation values. All eggs were studied together when the weekly number of eggs became less than 300. The egg hatching and counting protocol described previously was used to determine the field diapause incidence.

### Diapause termination

The diapause termination was also investigated in the field. Ovitraps being inappropriate to determine the beginning of the period of activity for the first generation of immature stages of the year, in 2011 and 2012 daily active surveys were conducted around the study area to find the first larvae hatchings (Fig 1). Ten places documented as infested by *A*. *albopictus* and localized in a five kilometer radius around the ovitraps network were inspected daily from March 1^st^. These places displayed unmovable breeding sites of varied nature (concrete blocks holes, stone containers, tree holes, etc.) and were maintained with the addition of water during dry conditions.

### Monitoring of mosquito population dynamics

The population dynamics was monitored weekly using a network of 15 ovitraps set up on June 21^st^ 2010 [34]. Ovitraps are artificial containers made up of 3 L black plastic buckets filled with 2 L of tap water changed weekly. In each container a floating polystyrene square (5 x 5 cm) is added to provide a support for oviposition. Ovitraps were hidden under vegetation and egg batches were collected weekly and brought back to laboratory for counting. The collection was stopped in November after two consecutive negative reports, marking the end of the active season of the Asian tiger mosquito. In 2011 and 2012 the network was reinstalled from the end of March and was expanded to 18 ovitraps. The surface of the trapping area was computed as the surface of the smallest polygon including all ovitraps with a buffer distance of 50 m, and covered 30.5 hectares in 2010 and 41.6 ha the following years. Both hatched and unhatched eggs of *A*. *albopictus* collected in ovitraps were counted in laboratory under a stereomicroscope Zeiss Stemi SV6.

#### Photo-thermal responses in population dynamics

The seasonal dynamics were studied according to the meteorological influence. The daily minimum (T_min_), maximum (T_max_) and average (T_mean_) air temperatures and daily precipitation were obtained from the Nice Côte d'Azur airport meteorological station, distant of 3 km from the study area (Fig 1). The daylight duration was provided by the “Institut de mécanique céleste et de calcul des éphémérides”. Daily data were pooled to calculate the weekly mean temperatures, total weekly precipitations and weekly mean daylight durations.

The development of poikilothermic organisms such as mosquitoes, being dependent of environmental temperature, the span of the mosquito life cycle should be analyzed according to the temperature range allowing its development. The time units (days) were reported to the number of accumulated daily degree (ADD), calculated as follows: ADD = ∑(((T_max_ + T_min_) / 2)—T_base_) with (T_base_) the critical temperature defined as the thermal threshold below which the development cannot occur, corresponding to 11°C [17,22].

The numbers of heat units required for different development times were calculated using demographic parameters of two strains. There are no differences in development rates between tropical and temperate strains, except in the embryonic stage [22]. Thus, we used development data from a tropical strain [35], completed with the embryonation rates–with or without diapause induction–of a local temperate strain [26] to calculate our day degree model.

### Statistical analyses

The diapause incidence percentages from laboratory and field eggs batches were modeled through sigmoid response curves fitted by day length using an iterative logistic model with a four parameters logistic regression. It allowed calculating the CPP for this population. The reaction norms were compared to determine the delay between maternal diapause induction and diapause initiation in offspring.

The threshold temperatures of the beginning of the egg laying activity and of the first seasonal egg hatching were determined by linear regressions of day length and the average minimum temperature of the week preceding egg laying activity.

Statistical analyses were performed using R 3.0.2 software [36].

## Results

### Diapause incidence

#### Experimental diapause incidence

**Fig 3:**
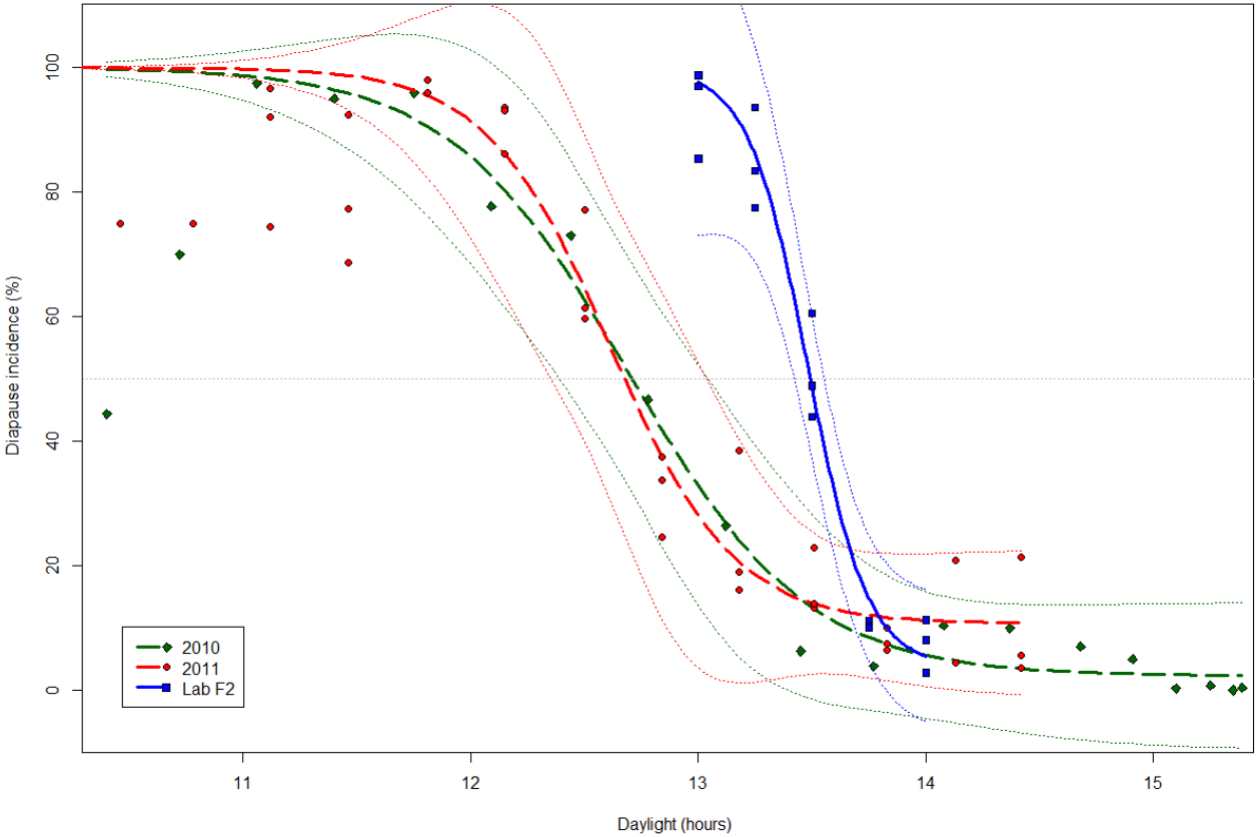
Photoperiodic analyses of egg diapause induction and initiation in *Aedes albopictus*. X axis represents the mean daylight duration during the egg laying week in the field in 2010 (green diamonds, n = 10) and 2011 (red dots, n = 36). Photoperiod inducing diapause was studied at fixed day length in laboratory (blue squares, n = 15). Sigmoid curves modeled for egg diapause incidences are in bold solid line (laboratory) or solid dashed lines (field). Smooth dotted lines represent the 95% confidence intervals. The sigmoid response curves ignore the field data points of decreasing diapause incidence for day length ≤ 11.5 hours, as these outliers are probably the result of a selection trend. The horizontal dot line (light grey) marks 50% of diapause incidence. The difference between blue curve (laboratory data) and green and red curves (field data) highlights the delay between maternal diapause induction and diapause preparation in offspring.

As expected, under fixed temperature and photoperiod in the laboratory, the complete shift in the number of eggs entering diapause incidence occurs between a day length of 13.0 and 14.0 hours. The CPP of this population is equal to 13.5 hours of light according to the sigmoid curve model of diapause induction (Fig 3).

#### Diapause incidence in the field

**Fig 4:**
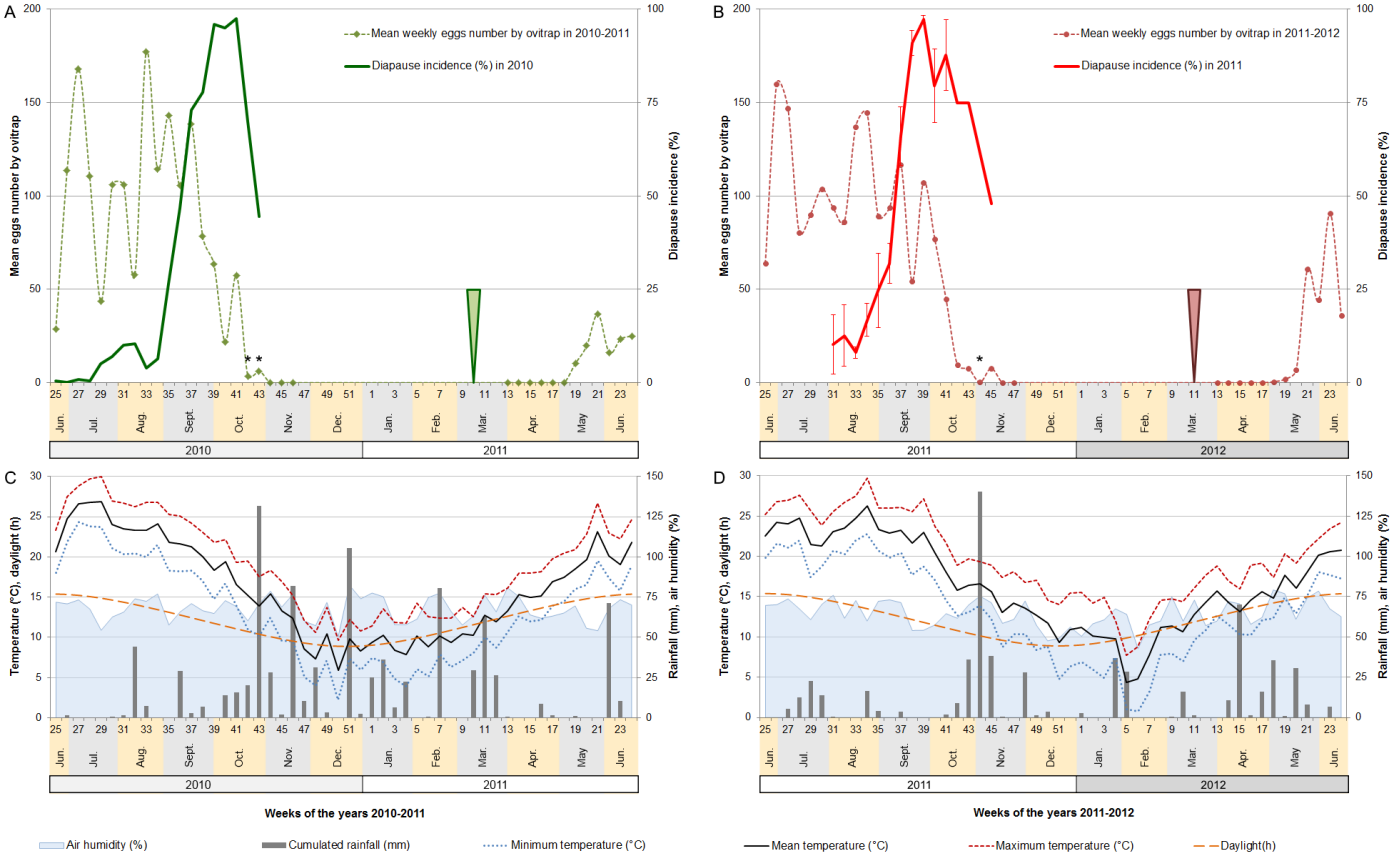
Seasonal profiles of oviposition activity and diapause timing of *Aedes albopictus* population (A,B) from summer solstice 2010 to summer solstice 2012 under the Mediterranean climatic conditions (C,D) of the city of Cagnes-sur-Mer, France. Asterisk represents positive samples with less than 100 eggs collected. Arrows show the week of sampling of the first larvae of the year.

The diapause incidence was compared to the average day length (Fig 3) of the week of field monitoring. The diapause rate showed a similar pattern between 2010 and 2011. It increases rapidly in the field during September (Fig 4A and 4B). Half of the eggs laid in the second week of September (week 36) were programmed to enter into diapause. The moment when 50% of the eggs initiate diapause corresponds to a day length of 12.7 hours (12h41), and occurred on September 11^th^. After the 39^th^ week, more than 95% of eggs laid were in prediapause stage, the diapause incidence stayed high for the first half of October. It decreased significantly from mid-October while the number of laid eggs decreased sharply.

#### Comparison of experimental and field diapause incidence

The experimentally determined CPP is of 13.5 hours; in the field this day length corresponds to the 25^th^ of August. The CPP is higher by 0.8 hour than the day length monitored in the field when embryonic diapause would be programmed in 50% of the eggs. This difference represented a time span of 17 days after the date of the CPP. It suggests the influence of environmental factors as well as a physiological delay during maternal diapause induction. The transition from a non-diapausing day length to a day length inducing the maximal diapause incidence should last theoretically 20 days (with the laboratory determined day lengths), but it lasted twice that time in the field.

#### Dormancy termination

The very first larvae were sampled both years in the first half of March (the 8^th^ in 2011 and the 13^th^ in 2012) and in the same breeding site (holes in concrete stud). A period of 25 (in 2010) to 26 weeks (in 2011) was needed between the time when 50% of eggs entered in diapause and the observation of the first viable hatched larva the following year. This period was similar from year to year despite different winter conditions (see below).

### Seasonal pattern of oviposition

A total of 53790 eggs of *A*. *albopictus* were collected during the field samplings (21126 eggs during the period between the summer solstices of 2010 and 2011, and 32664 during the period between the summer solstices of 2011 and 2012s). A mean density of 68.4 ± 49.0 eggs per ovitrap per week was collected from weeks 19 to 45 in 2011. All the ovitraps were found positive during 14 weeks in 2010 and 12 weeks in 2011, between June 27^th^ and November 2^nd^. The egg laying activity highly increased after the summer solstices of both years and remained globally high despite variations until the second half of September (Fig 4A and 4B). After that, the mean number of eggs per ovitrap sharply decreased. No egg laying activity was detected after November 2^nd^ in 2010 and November 14^th^ in 2011 (data already published in [34]).

The first eggs laid by the first annual generation of adults were detected in the first half of May for both years. Taking into account the date of first egg hatchings, it corresponds to a development length of 9 weeks in 2011 and of 7 weeks in 2012.

#### Photo-thermal responses in population dynamics

The elapsed time at different phases of the diapause process was converted in ADD above 11°C, the estimate thermal units available for mosquito development (Table 1). We considered here the 11^th^ day of September as the generic day of diapause initiation, with 50% of the eggs laid in diapause. Diapause was induced in 50% of eggs 15 days before the date of diapause initiation (Figs 3, 4A and 4B). In the field the fortnight elapsed between these estimated dates correspond to 205 to 225 ADD. It is more than the heat units necessary in *A*. *albopictus* for the development from pupae to first egg laying, or from pupae to the end of the second gonotrophic cycle. The photothermic analysis of the prediapause stage suggests that diapause was not completely induced in field eggs of the first gonotrophic cycle. This is corroborated by the laboratory experiment, where females exposed to short photoperiod during at least 11 days show a switch to complete induction of diapause (S1 File).

**Table 1:** Comparison of theoretical and field observed development time of *Aedes albopictus* stages using a day-degree model. The thermal threshold is equal to 11°C.

Development time	Theoretical ADD	Observed ADD (year)
From egg hatching to first oviposition	245^a^	
From pupation to first gonotrophic cycle end	142^b^	
From pupation to second gonotrophic cycle end	198^b^	
Mean generation (without diapause)	295^c^	
Mean generation (with diapause)	310^c^	
From CPP to diapause initiation		205 (2010)^b^
		225 (2011)^b^
From CPP to the end of the oviposition activity		558 (2010)^c^
		729 (2011)^c^
From diapause initiation to the first spring hatching		424.85 (2010–2011)^d^
		615.5 (2011–2012)^d^
From the first spring hatching to the first oviposition		270 (2011)^a^
		201 (2012)^a^

^a,b,c,d^: data compared against each other’s.

For both years, from the date when field day length was equal to the CPP to the end of the monitored oviposition activity, there were actually more heat units available than required to complete one generation of *A*. *albopictus* with effective induction of diapause in the laid eggs. The sum of heat units available even allowed the complete development of 2 generations in 2011 (605 ADD necessary).

The 2010–2011 winter was rainier than the 2011–2012 winter (324.1 mm versus 85.3 mm of cumulated rainfalls) (Fig 4C and 4D). The average mean temperature was similar between both winters (9.66 ± 1.78°C for the 2010–2011 winter versus 9.60 ± 2.85°C for the 2011–2012 winter), however the last winter was more contrasted: it started hot and dry and followed by two cold weeks with 8 days of negative minimal temperature. Consequently, from the generic day of diapause initiation to the date of observation of the first larva hatching, there was 424.85 ADD during the winter 2010–2011 and 615.5 ADD during the winter 2011–2012. This represents an increase of 45% ADD_._

The two days following the observation of the first larva in 2011 were the last days of spring where the mean temperature was less than 10°C (S2 Fig) with a minimum temperature less than 5°C. In 2012 such low temperatures ended a week before the observation of the first larva. Only 2 days of chilling with T_mean_ ≤ 5°C were encountered in both winters. These winters differed little in their average temperature: the 2010–2011 winter was 1.3°C colder than the 2011–2012 winter, with respectively 73 days versus 38 days under an average temperature of 10°C. Only the 2011–2012 winter encountered a freezing period with 9 days of T_min_ ≤ 0°C. However when looking at the cumulated thermal degree units available for *A*. *albopictus*, both winters differed a lot. Indeed, the 2011–2012 winter had 45% more ADD than the winter 2010–2011. As the first annual larvae appeared quite simultaneously both years despite different winter meteorological conditions, other factors may be involved. The day length corresponding to the first egg hatching was equal or superior to 11.5 hours.

The average minimum temperature of the week preceding egg laying activity is estimated with an intercept of the linear regression at 12.5°C. From egg hatching to the first oviposition, the development time measured in 2011 in the field corresponded closely to the heat units necessary (245 ADD). However in 2012, only 201 ADD were observed between the monitored hatching and egg laying; a correspondence in ADD measured is observed when considering the time where the number of eggs laid reached 5% of the annual weekly mean number of eggs laid.

## Discussion

### Diapause induction

The CPP is under polygenic control, evolving with latitude [28], and is a very plastic life history trait sensitive to high temperature [37] and larval nutritive state [37]. The observed difference in the field of 0.8 hour between the CPP and the diapause preparation into 50% of the eggs is important and could be partly explained by environmental conditions. Indeed, individuals of the natural population are exposed to fluctuating temperatures, which can slightly interact with the photoperiodic signal. In the northern tamarisk beetle *Diorhabda carinulata*, high daily thermal amplitude was demonstrated to reduce the CPP by 0.15 hour for laboratory controlled amplitude of 10°C [38]. This kind of effect could exist in *A*. *albopictus*, however the median daily thermal amplitude did not reach 6.7°C during the month preceding and following the day of CPP. Furthermore, the effects of thermal amplitude in the field should be counterbalanced by the broad variability of larval nutrition; indeed, adults starved at larval stages showed a CPP lengthened up to 0.25 hour [37]. The different temperature and food conditions experienced by individuals do not entirely explain the difference in photoperiodic responses between the field and the laboratory populations. The photothermic analysis of the prediapause stage suggested diapause was not completely induced in eggs of the first gonotrophic cycle in the field. Indeed, there was enough ADD during this time for sensitive stages to develop from the pupae to the end of the second gonotrophic cycle (Table 1). This supposition is corroborated by laboratory experiment at 21°C on this strain, showing a switch to complete induction of diapause in eggs of females exposed to short photoperiod during at least 11 days (S1 File). A similar observation was made on North America strains [25]. Thus diapause induction should only be considered fully effective for eggs from the second gonotrophic cycle. As a physiological process, diapause induction could occur slightly faster in the field where mean temperatures are higher by 1 to 3°C (Fig 4C and 4D).

Egg diapause must be induced at the appropriate time to obtain an initiation accurately synchronized with seasonal conditions. In the field, the maximum diapause incidence occurred a month before the cooling of the minimal temperature under 15°C (Fig 4). After diapause induction it is essential that the environment remains sufficiently warm to allow for the completion of at least one full generation of *A*. *albopictus* progenitors and of a diapausing offspring [39]. The sum of heat units recorded both years in our field study allowed the complete development of one, and sometimes two generations of progenitors. Does it mean the CPP of this Asian tiger mosquito population is not perfectly adapted to the local Mediterranean environment? Even though it is not possible to assert this with certainty, the data are consistent with a “bang-bang” strategy [40]: during the favorable period mosquitoes reproduce and do not enter diapause, up to a specific date, illustrated as the CPP, when all the organisms will have to initiate diapause. The apparent precocity of prediapause stage could then be explained by the overlapping generations of *A*. *albopictus* and the stochasticity of rainfalls occurrence or intensity during Mediterranean summers.

Most of the population enters diapause at the end of September, however a decrease of the diapause incidence is observed in eggs laid after that date on both years. This trend is not directly induced by the photoperiod, as the diapause incidence of our local temperate populations in the laboratory remains higher than 90% for rearing day lengths of 13.0 as well as 9.0 hours [26]. As diapausing eggs are not sensitive to environmental stimuli, only non-diapausing eggs can hatch to produce a new generation of breeders at the end of the season. Thus the overall decrease in the number of active *A*. *albopictus* at end of the season–decrease caused by the initiation of diapause–leads to a selection of phenotypes that express less or no diapause in the remaining active population. Under favorable climatic conditions this selection phenomenon allows for a rapid decline of diapause in the population, such as documented in previous studies: In Rome, Italy, the minimum egg hatching rate obtained after a field sampling of *A*. *albopictus* eggs realized in the fall of year 2000 was 17%—as compared to less than 5% in our study–meaning that a significant part of the *A*. *albopictus* population had not entered diapause [41]. In southernest's cities of Spain, a small part of the Asian tiger mosquito population is probably unable of winter diapause [42,43]. This phenomenon is even more evident in North America where a progressive selection of the non-diapausing phenotypes could explain that in 2011 more than 60% of eggs laid by the strain of Miami are not in diapause [44,45]. The non-diapausing individuals must be advantaged in areas with warm winters, having the exclusive benefit of a longer period of time for reproduction and proliferation.

In metropolitan France, non-diapausing phenotypes are probably eliminated afterwards by winter conditions. Indeed, in our study the complete end of the egg laying period occurred at the same time as the most important rainfalls of the year (Fig 4). These exceptional rainfall events occurring on week 44 may have precipitated the end of the active season of the Asian tiger mosquito by causing an important mortality of the immature stages [46] and remaining adults. However, a continuous residual egg laying activity was observed during the winter in Rome [47] and in southern Spain [42,43], but none was reported in other southern cities, such as Athens, Greece [48] or Tirana, Albania [49] where the oldest European populations of the Asian tiger mosquito are found. In Rome (41°53′N), despite repeated flooding during winter the eggs remained unhatched, thus confirming their diapause status and leading to the strong assumption that they had been laid by long-lived *A*. *albopictus* females of the last seasonal generation [47]. The anthropogenic modification of the environment is likely to be involved in the winter survival of mosquitoes [50]. However in France, based on a 6 years long population sampling—winter monitoring included -, the persistence of adult *A*. *albopictus* populations during winter seems unlikely to happen soon [34].

### Dormancy termination

The beginning of diapause termination in the field was estimated in our study by a monitoring of egg hatching, although this method cannot discriminate the termination *per se* of the post-diapause quiescence. This quiescence follows diapause termination until environmental conditions become favorable. The monitored breeding sites were flooded and maintained with water by the investigator, therefore only eventual low temperatures would have sustained quiescence and have substantially delayed egg hatching. Based on our study, and according to previous field observations [41] and models [18], the values for the hatching onset parameters correspond to a spring temperature above 10.5°C and a spring photoperiod above 11.25 hours. The termination of diapause leads to a gradual reactivation of eggs in the field. The factors involved in this process where studied in laboratory and differed with regards to the mosquito species [27]. Mainly, a long-term exposition at chilling but not freezing temperature triggers egg reactivation. Photoperiodism is also effective, long day treatment having a strong reactivation effect in eggs of *A*. *caspius* and *A*. *triseriatus*. The adaptive advantage of photothermic interaction is obvious in the field, photoperiodism preventing early and inappropriate hatchings under changing temperatures during winter. Our data are consistent–but not conclusive–with a reactivation dependent of a photothermic interaction. The estimation of the average minimum temperature of the week preceding egg laying activity found in this study is inferior by 0.5°C from the 13°C found in northern Italy on adult female emergence [51]. As some precocious egg laying and adults were observed in 2012, earlier undetected hatchings could explain this discrepancy between monitored and development time. However, there occurrence resulted more likely from an accelerated larval development in breeding sites warmer than field conditions, such as rain barrels exposed to the sun.

### The long generation

**Fig 5:**
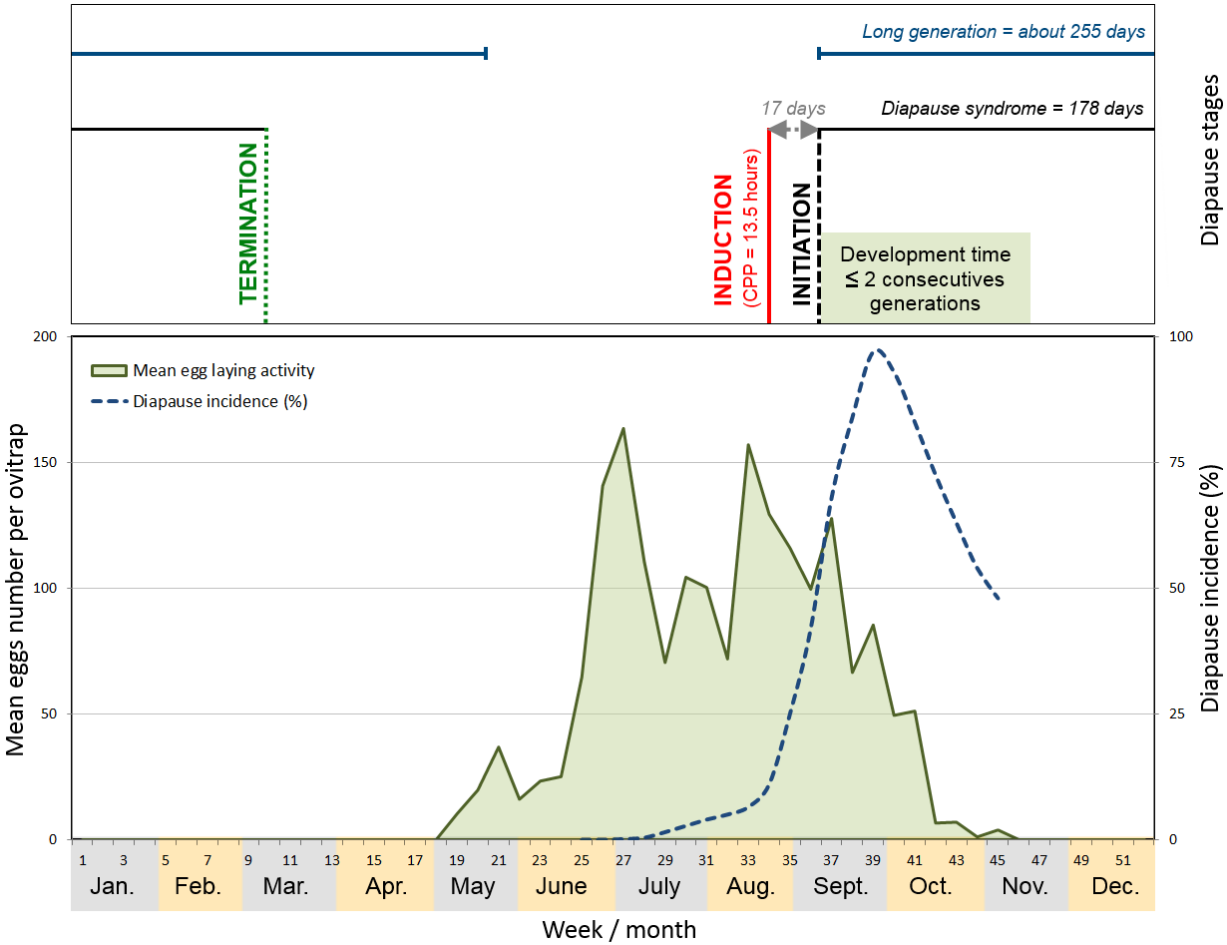
Schematic depiction of the seasonal activity and diapause process of *Aedes albopictus* population in the Mediterranean city of Cagnes-sur-Mer, France. Horizontal lines represent the mean durations of diapause syndrome and of the long generation of *A*. *albopictus*. Vertical lines delineate the moment when diapause is induced (in red) or initiated (in dark) in 50% of eggs, and the start of diapause termination (in green).

Maternal induced egg diapause plays a central role for the wintry long generation, with phases studied here in a field population: from the induction through to the diapause initiation, and following up to diapause termination (Fig 5). The first annual generation spends a long time in immature stages, with 20 to 26 weeks at the egg stage and about 7 weeks at the larval stages. This long development time represents for mosquito control operators a great opportunity to thwart the species population dynamics by targeting the eggs and larvae of a unique generation. The mechanical suppression of breeding containers where eggs have accumulated as a result of female seasonal oviposition choice during the fall [52] would be an especially efficient procedure for that purpose. As mosquito control operators cannot contact every inhabitants living in the suburban areas where the major biting discomfort is experienced, the main issue to overcome, particularly in a period with no adult nuisance, will deal with communication aiming to modify community habits [53].

When compared to models based on Medlock et al. [54], the time span of mosquito activity measured in our study is already longer than projections for the current period [55] and even equal to projections for 2030–2050 [18]. In these models, the Mediterranean area is expected to be very vulnerable to climatic change, with a strong overall warming–including a temperature increase of 2–4°C during fall and winter up to the years 2071–2100 –and a reduction in precipitation [56]. These models slightly underestimate the extent of the growing season in urban areas, where temperature is generally higher than in natural environments. Consequently, an upward revision of the estimated period of activity of *A*. *albopictus* should be considered for cities. The distribution area, the extent of the growing season and developmental rates are all expected to increase due to global warming. It would notably make possible the production of one or two additional yearly generations [57]. The major biological response to climate change in mosquitoes is expected to be an adaptation of the diapause induction and termination to adjust to shorter winters [23].

## Conclusions

The phenological population dynamics of *A*. *albopictus* was studied in the light of the major role played by the evolutionary process of diapause. The photoperiodic data of diapause induction, initiation and termination of the population of Asian tiger mosquito will serve as reference for future work on the evolution over time and dissemination of this invasive species, with regards to local adaptation and global warming. This study provides a basis of field and phenological data that will be useful to apprehend the future expansion of *A*. *albopictus* population toward Northern Europe, and to develop temporal abundance models in order to simulate and test innovative strategies for vector control operators.

## Supporting Information

S1 Fig Egg hatched count is a more reliable method than larval count, the latter being sensitive to mortality of first instar larvae. (DOCX)

S2 Fig Daily temperature during the period of diapause termination in spring 2011 and 2012 in Nice, France. (DOCX)

S1 File Time requirement for maternal induction of diapause in *Aedes albopictus*. (DOCX)

S1 Table Geographic coordinates (system WGS84) of ovitraps and breeding sites used for the monitoring of population dynamics of *Aedes albopictus* from 2010 to 2012 around Cagnes-sur-Mer, France. (DOCX)
