# Supplementary material for: Seasonal Synchronization of Diapause Phases in Aedes albopictus (Diptera: Culicidae)
Source: PLoS One. 2015 Dec 18;10(12):e0145311. doi: 10.1371/journal.pone.0145311 (PMC4686165; doi:10.1371/journal.pone.0145311)
Supplement: S1 Fig — (DOCX) [file pone.0145311.s001.docx]

**S1 Fig. Egg hatched count is a more reliable method than larval count, the latter being sensitive to mortality of first instar larvae.** Independent batches of *Aedes albopictus* eggs (n>300) were stimulated during 3 hours with ascorbic acid to hatch. Eggs were removed, dried, and then live first instar larvae (A) and hatched eggs (B) were counted.
