# Supplementary material for: Seasonal Synchronization of Diapause Phases in Aedes albopictus (Diptera: Culicidae)
Source: PLoS One. 2015 Dec 18;10(12):e0145311. doi: 10.1371/journal.pone.0145311 (PMC4686165; doi:10.1371/journal.pone.0145311)
Supplement: S2 Fig — (DOCX) [file pone.0145311.s002.docx]

**S2 Fig. Daily temperature during the period of diapause termination in spring 2011 and 2012 in Nice, France.** Value (dotted line) and linear regression (solid line) of daily mean temperature during the 3 weeks preceding and following the first annual hatching of 2011 (in blue) and 2012 (in red).
