# Supplementary material for: Seasonal Synchronization of Diapause Phases in Aedes albopictus (Diptera: Culicidae)
Source: PLoS One. 2015 Dec 18;10(12):e0145311. doi: 10.1371/journal.pone.0145311 (PMC4686165; doi:10.1371/journal.pone.0145311)
Supplement: S1 File — (DOCX) [file pone.0145311.s003.docx]

**S1 File: Time requirement for maternal induction of diapause in *Aedes albopictus*.**

In order to determine the duration under SD the female must be exposed to, to induce diapause in her eggs, long-days reared larvae of SPAM strain were transferred under short-day exposure at the moment of pupation. Thirty newly emerged females were put in cage with 30 males under SD with 10% sucrose solution. Cages were transferred under LD exposure between 5 and 8 days after female emergence and provided with a blood meal on anesthetized guinea pig and an oviposition site. Batches of eggs from oviposition sites were collected 7 days after the blood meal and maintained in incubator in darkness at 21°C and 70% RH. Three replicates of at least 400 10-days old eggs were immerged in over-oxygenate tap water, and with an addition of 100 mg of ascorbic acid per liter of water which is a strong hatching stimulus. This hatching protocol is repeated next day to make sure of having real hatching rate. Unhatched eggs are considered in diapause if embryos are fully developed without deformity. The presence of ocelli and egg burster (a spine on the clypeus of embryo by which it breaks the egg chorion in hatching) on embryos is inquired into eggs decolorized with Trpiš solution. The duration of SD exposure needed for a female to induce diapause in her eggs **(Figure A)** is deducted by the egg hatching rate, calculated with embryonated and hatched eggs:

Hatch % = (hatched eggs x 100) / (embryonated unhatched eggs + hatched eggs)

**Figure A. Percentage of egg hatching in function of the number of short days underwent by the adult mother of a temperate strain of *Aedes albopictus* at 21°C.** The SD exposure began at pupae stage. Horizontal axe indicate the day of switch from diapausing short days (9L:15D) to long days (16L:8D) imaginal exposure. An exposure of adult females during 7 days under a short day photoperiod from the end of the pupal stage to the first blood meal is required to induce diapause in all her progeny. Taking account a mean duration of 4 days under pupal stage, 11 days under short photoperiod during photosensitive stages induce more than 90% of diapause. Interestingly, same induction duration was found for the North-American INDY strain of *A. albopictus* [23], suggesting that the induction duration is similar for temperate strains.
