# Supplementary material for: Seasonal Synchronization of Diapause Phases in Aedes albopictus (Diptera: Culicidae)
Source: PLoS One. 2015 Dec 18;10(12):e0145311. doi: 10.1371/journal.pone.0145311 (PMC4686165; doi:10.1371/journal.pone.0145311)
Supplement: S1 Table — (DOCX) [file pone.0145311.s004.docx]

| **Type** | **Code** | **Latitude (decimal degrees)** | **Longitude (decimal degrees)** |
| --- | --- | --- | --- |
| Breeding site | **A** | 43.67742194 | 7.20158278 |
| Breeding site | **B** | 43.66843000 | 7.21472083 |
| Breeding site | **C** | 43.64094389 | 7.13159000 |
| Breeding site | **D** | 43.66993306 | 7.14489583 |
| Breeding site | **E** | 43.67124806 | 7.19998972 |
| Breeding site | **F** | 43.66417889 | 7.19090583 |
| Breeding site | **G** | 43.68328889 | 7.15502389 |
| Breeding site | **H** | 43.66431000 | 7.16542694 |
| Breeding site | **I** | 43.65548389 | 7.15819083 |
| Breeding site | **J** | 43.66614889 | 7.15972278 |
| Ovitrap | **1** | 43.66408056 | 7.17061944 |
| Ovitrap | **2** | 43.66246111 | 7.16865556 |
| Ovitrap | **3** | 43.66274722 | 7.16825278 |
| Ovitrap | **4** | 43.66428056 | 7.16549722 |
| Ovitrap | **5** | 43.66444167 | 7.16582222 |
| Ovitrap | **6** | 43.66542778 | 7.16074722 |
| Ovitrap | **7** | 43.66565556 | 7.16053056 |
| Ovitrap | **8** | 43.66048333 | 7.16169444 |
| Ovitrap | **9** | 43.66066667 | 7.16134444 |
| Ovitrap | **10** | 43.66696389 | 7.15885833 |
| Ovitrap | **11** | 43.66681111 | 7.15902778 |
| Ovitrap | **12** | 43.66843333 | 7.16146389 |
| Ovitrap | **13** | 43.66598889 | 7.16161667 |
| Ovitrap | **14** | 43.66678333 | 7.16015833 |
| Ovitrap | **15** | 43.66896389 | 7.15858611 |
| Ovitrap | **16** | 43.66992778 | 7.16303611 |
| Ovitrap | **17** | 43.66914444 | 7.16206944 |
| Ovitrap | **18** | 43.66316667 | 7.16789167 |

**S1 Table. Geographic coordinates (system WGS84) of ovitraps and breeding sites used for the monitoring of population dynamics of *Aedes albopictus* from 2010 to 2012 around Cagnes-sur-Mer, France.**
